# f-Element silicon and heavy tetrel chemistry

**DOI:** 10.1039/d0sc04655h

**Published:** 2020-09-24

**Authors:** Benjamin L. L. Réant, Stephen T. Liddle, David P. Mills

**Affiliations:** Department of Chemistry, School of Natural Sciences, The University of Manchester Oxford Road Manchester M13 9PL UK steve.liddle@manchester.ac.uk david.mills@manchester.ac.uk

## Abstract

The last three decades have seen a significant increase in the number of reports of f-element carbon chemistry, whilst the f-element chemistry of silicon, germanium, tin, and lead remain underdeveloped in comparison. Here, in this perspective we review complexes that contain chemical bonds between f-elements and silicon or the heavier tetrels since the birth of this field in 1985 to present day, with the intention of inspiring researchers to contribute to its development and explore the opportunities that it presents. For the purposes of this perspective, f-elements include lanthanides, actinides and group 3 metals. We focus on complexes that have been structurally authenticated by single-crystal X-ray diffraction, and horizon-scan for future opportunities and targets in the area.

## Abstract 2

In this perspective we review the molecular chemistry of f-element silicon and heavy tetrel complexes.

## Graphical abstract

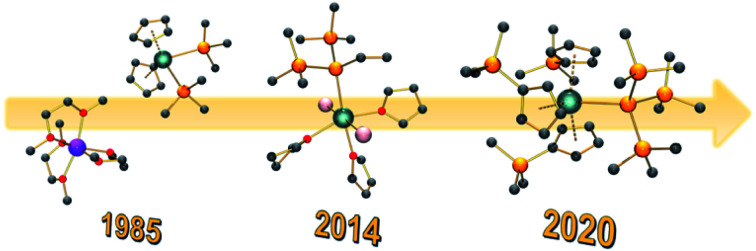

## Introduction

**Fig. 1:**
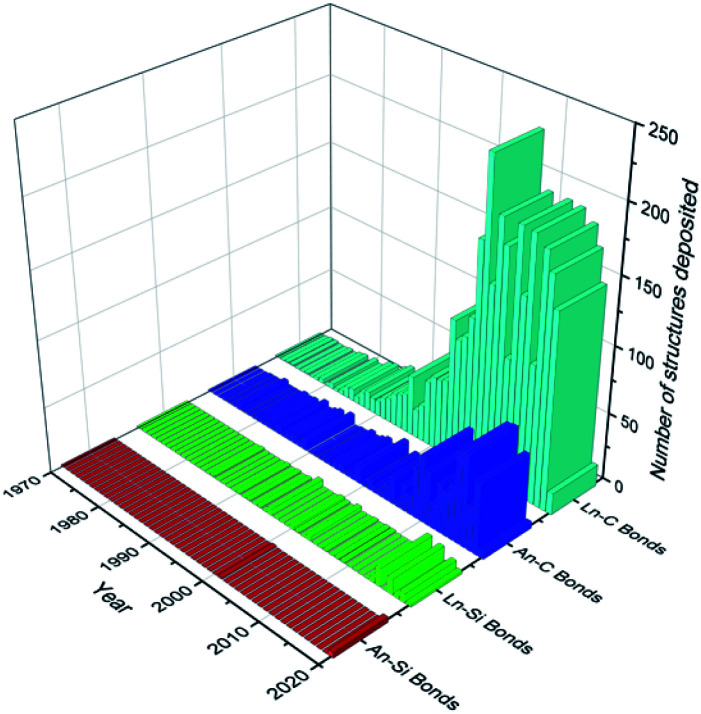
Graph depicting the number of structures deposited into the CCDC per year (as of August 2020) for Ln/An–C/Si σ-bonds.^24^ Total figures: Ln–C = 3049, Ln–Si = 58, An–C = 653, An–Si = 5.

Recent decades have seen a rapid growth in the development and study of f-elements for a wide variety of applications including catalysis,^1,2^ imaging,^3,4^ data storage^5,6^ and nuclear fuel.^7,8^ The chemistry of the f-elements, the lanthanides (Ln; defined here to include the group 3 metals scandium, yttrium and lanthanum as well as cerium to lutetium inclusive) and actinides (An), is defined by predominantly ionic bonding regimes, high coordination numbers, and a preference for hard f-element cations to bond with hard bases (by the HSAB hard–soft–acid–base definition). As such, the aqueous solution chemistry of the f-elements is dominated by compounds containing essentially electrostatic bonds between the f-element ion and the most electronegative p-block elements (*e.g.* halides, O, N).^9^ Non-aqueous techniques have facilitated the recent expansion of f-element heavy chalcogen and pnictogen chemistry;^10–13^ f-element carbon chemistry is now mature, with a wide variety of ligand types extensively reviewed including alkyls,^14,15^ carbenes,^16–18^ cyclopentadienyls^19,20^ and arenes.^21–23^ However, the f-element solution chemistry of heavier tetrels has remained in the shadow of the lightest member carbon; this is exemplified by analysis of the number of crystallographically characterised examples of f-element carbon and silicon σ-bonds that have been deposited with the Cambridge Crystallographic Data Centre (CCDC) as of August 2020 (Fig. 1). The contrast is even greater upon descent of Group 14, with only a handful of examples of structurally authenticated complexes of f-elements bonded to germanium (4 examples), tin (11 examples) or lead (2 examples) in total.^24^ The paucity of f-block-heavier tetrel linkages is representative of the chemistry of these elements being less developed than the d-transition metals (TM);^9,25^ this is also exemplified by a comparative dearth of f-element–metal bonds.^26^

Although interest in f-element silicon chemistry has started to grow in recent years, the current number of f-element–silicon bonds reported (63 examples) is comparable to the total number of f-element–carbon bonds that were reported by 1986.^24^ The most investigated application of complexes containing M–Si bonds to date is the (hydro)-silylation of unsaturated hydrocarbons; TM complexes have been shown to promote this chemistry for decades, and attention has turned to the f-block for comparative studies.^27–30^ Also of significance is the potential application of uranium silicides (*e.g.* U_3_Si_2_, USi_2_) as alternatives to conventional UO_2_ fuel due to an increase in uranium density and a larger thermal conductivity in the former materials, potentially allowing prolonged generation of energy from nuclear fuels.^31–33^ Given historical experimental and computational limitations, early reports of f-element silicon chemistry lack in-depth analysis of the bonding and properties of the M–Si linkage compared to what can be done now; a higher level of analysis has only started to be practicable recently and these fundamental studies are the necessary first step for this field to develop further. The increasing rate of development in f-element silicon chemistry in the last two decades has provided the motivation for this perspective; the heavier tetrels are discussed herein to inspire the future development of the f-element chemistry of these elements also, but silicon remains the focus.

A silicon donor atom is commonly introduced to an f-element metal centre in one of two ways: (1) salt elimination/metathesis of a group 1 or 2 metal silanide anion (SiR_3_^−^) transfer agent (Group 11 and 12 metal silanides are also known but are less commonly used) with an f-element halide-precursor to produce a polarised-covalent f-element tetrel linkage; or, (2) dative coordination of a neutral Si(ii) silylene reagent to form an adduct with an f-element complex that has a vacant coordination site, or, where these sites can be generated *in situ* by the displacement of weakly bound donor solvent molecules. The chemistry of both of these silicon reagent families have previously been reviewed.^34–36^ Examples of f-element silylene adducts are relatively scarce, and f-element silanide complexes are dominated by the tris-(trimethylsilyl)silanide anion, ({Si(SiMe_3_)_3_})^−^, frequently referred to as hypersilanide, and its derivatives.

This perspective highlights work reported in the field of f-element silicon and heavier tetrel chemistry to August 2020, focusing on structurally characterised examples Table 1. Our aims are to show the state-of-the-art in the field, the limitations of our current understanding, and to inspire researchers to develop and progress f-element silicon chemistry more rapidly in future. This perspective is split into four sections, with separate components on Ln(ii)–, Ln(iii)–, and An–Si chemistry, and a final section on f-element heavy tetrel chemistry, with subsections for ease of reference.

**Table 1:** F-Element tetrel complexes in this perspective

Complex name	Complex number	M–E distance/Å	Ref.
**M–Si complexes**			
[Yb(SiPh_3_)_2_(THF)_4_]	**1**	3.158(2)	40
[Sm{Si(SiMe_3_)_3_}_2_(THF)_3_]	**2-Sm**	3.1716(11)	41
[Eu{Si(SiMe_3_)_3_}_2_(THF)_3_]	**2-Eu**	3.1766(17)	41
[Yb{Si(SiMe_3_)_3_}_2_(THF)_3_]	**2-Yb**	3.0644(7)^a^	41
[Sm{[Si(SiMe_3_)_2_SiMe_2_]_2_}(THF)_4_]	**3-Sm**	3.2288(14)	41
[Eu{[Si(SiMe_3_)_2_SiMe_2_]_2_}(THF)_4_]	**3-Eu**	3.2105(18)^a^	41
[Yb{[Si(SiMe_3_)_2_SiMe_2_]_2_}(THF)_4_]	**3-Yb**	3.1385(4)^a^	41
[Yb{Si(SiMe_2_H)_3_}_2_(THF)_3_]	**4**	3.011(3)	42
[Sm{[Si(SiMe_3_)_2_]_2_SiMe_2_}(THF)_4_]	**5-Sm**	3.1779(15)^a^	44
[Yb{[Si(SiMe_3_)_2_]_2_SiMe_2_}(THF)_4_]	**5-Yb**	3.0615(18)	44
[Sm{Si(SiMe_3_)_2_SiMe_2_}(THF)]_2_	**6**	3.1438(11)^a^	44
[Sm{[Si(SiMe_3_)_2_SiMe_2_]_2_O}(THF)_3_]	**7-Sm**	3.1650(17)^a^	44
[Yb{[Si(SiMe_3_)_2_SiMe_2_]_2_O}(THF)_3_]	**7-Yb**	3.0694(11)^a^	44
[Yb{[Si(SiMe_3_)_2_SiMe_2_]OMe}_2_(THF)]	**8**	3.055(3)^a^	44
[Eu{Si(SiMe_3_)_2_Si(Me)[{OCH_2_CH_2_}_2_NMe]}_2_]	**9-Eu**	3.157(2)	44
[Yb{Si(SiMe_3_)_2_Si(Me)[{OCH_2_CH_2_}_2_NMe]}_2_]	**9-Yb**	3.050(3)	44
[Yb{Si(SiMe_3_)_2_Si([OCH_2_CH_2_]_3_N)}(THF)_2_]	**10**	3.025(4)	45
[Yb(Cp*){Si(SiMe_3_)_3_}(THF)_2_]	**11**	3.032(3)	46
[Sm(Cp*)_2_(SiH_3_){K(THF)}]_*n*_	**12-Sm**	––b	47
[Eu(Cp*)_2_(SiH_3_){K(THF)}]_*n*_	**12-Eu**	3.239(3)	47
[Yb(Cp*)_2_(SiH_3_){K(THF)}]_*n*_	**12-Yb**	3.091(3)	47
[Yb{Si(SiMe_3_)_3_}{μ-N(SiMe_3_)_2_}_2_K]	**13**	3.039(2)	48
[Sm(Cp*)_2_{Si(N^*t*^BuCH)_2_}]	**14**	3.191(1)	51
[Sm(Cp*)_2_{Si(O^*t*^Bu)[(N^*t*^Bu)_2_CPh]}]	**15**	3.4396(15)	53
[Sm(Cp*)_2_{Si(OC_6_H_4_-2-^*t*^Bu)[(N^*t*^Bu)_2_CPh]}]	**16**	3.3142(17)	53
[Eu{C_5_H_3_N[2,6-{NEt[Si{(N^*t*^Bu)_2_CPh}]}{N(SiMe_3_)_2_}_2_]	**17-Eu**	3.284(2)	54
[Yb{C_5_H_3_N[2,6-{NEt[Si{(N^*t*^Bu)_2_CPh}]}{N(SiMe_3_)_2_}_2_]	**17-Yb**	3.175(2)	54
[Yb{C_5_H_3_N-2-NEt,6-{NEt[Si{(N^*t*^Bu)_2_CPh}]}{N(SiMe_3_)_2_}_2_]	**18**	3.0426(15)	54
[Sc(Cp*)_2_{SiH(SiMe_3_)_2_}]	**19-Sc**	2.832(2)^a^	57
[Y(Cp*)_2_{SiH(SiMe_3_)_2_}]	**19-Y**	––b	56
[Nd(Cp*)_2_{SiH(SiMe_3_)_2_}]	**19-Nd**	––b	55,56
[Sm(Cp*)_2_{SiH(SiMe_3_)_2_}]	**19-Sm**	3.052(8)	55,56
[Nd(C_5_Me_4_Et)_2_{SiH(SiMe_3_)_2_}]	**20-Nd**	––b	56
[Sm(C_5_Me_4_Et)_2_{SiH(SiMe_3_)_2_}]	**20-Sm**	––b	56
[Sc(Cp*)_2_{SiH_2_(SiPh_3_)}]	**21**	2.797(1)	57
[Sc(Cp)_2_{Si(SiMe_3_)_3_}(THF)]	**22**	2.862(3)	58
[Lu(Cp*)_2_{SiH_2_(*o*-MeOC_6_H_4_)}]	**23**	2.823(5)	59
[Y{Si(SiMe_3_)_2_Et}(I)_2_(THF)_3_]	**24-Y**	2.9613(18)	60
[Gd{Si(SiMe_3_)_2_Et}(I)_2_(THF)_3_]	**24-Gd**	2.989(2)	60
[Y{Si(SiMe_3_)_3_}(I)_2_(THF)_3_]	**25-Y**	2.979(3)	60
[Gd{Si(SiMe_3_)_3_}(I)_2_(THF)_3_]	**25-Gd**	––b	60
[Sm_3_Cp*_6_(μ-Si_2_H_4_)(μ-SiH_3_)]	**26A**	2.954(2)–3.174(4)	61
[Sm_3_Cp*_6_(μ-SiH_3_)_3_]	**26B**	3.134(6)–3.155(3)^c^	61
[Sm_3_Cp*_6_(μ-Si_3_H_6_)(μ-SiH_3_)]	**26C**	––c	61
[Tm{AlMe_2_(η^5^-NC_4_Me_4_)_2_}(AlMe_3_)(μ-CH_2_)(μ-SiH_3_)(AlMe_2_){AlMe_2_(NC_4_Me_4_)}]	**27**	2.574(11)/3.087(6)^c^	63
[Y{Si(SiMe_2_H)_3_}_2_(OEt_2_)(μ^2^-Cl)_2_(μ^3^-Cl)K_2_(OEt_2_)_2_]_∞_	**28**	3.035(1)	42
[Li(DME)_3_][Dy(Cp)_2_(SiMe_3_)_2_]	**29-Dy**	––b	65
[Li(DME)_3_][Ho(Cp)_2_(SiMe_3_)_2_]	**29-Ho**	––b	65
[Li(DME)_3_][Er(Cp)_2_(SiMe_3_)_2_]	**29-Er**	––b	65
[Li(DME)_3_][Tm(Cp)_2_(SiMe_3_)_2_]	**29-Tm**	––b	65
[Li(DME)_3_][Lu(Cp)_2_(SiMe_3_)_2_]	**29-Lu**	2.888(2)	64–66
[{K(18-crown-6)}_2_Cp][Sm(Cp)_2_{[Si(SiMe_3_)_2_SiMe_2_]_2_}]	**30-Sm**	3.056(4)^a^	67
[{K(18-crown-6)}_2_Cp][Gd(Cp)_2_{[Si(SiMe_3_)_2_SiMe_2_]_2_}]	**30-Gd**	3.0277(20)^a^	53
[{K(18-crown-6)}_2_Cp][Tb(Cp)_2_{[Si(SiMe_3_)_2_SiMe_2_]_2_}]	**30-Tb**	3.0189(26)^a^	53
[{K(18-crown-6)}_2_Cp][Ho(Cp)_2_{[Si(SiMe_3_)_2_SiMe_2_]_2_}]	**30-Ho**	2.9925(24)^a^	53
[{K(18-crown-6)}_2_Cp][Tm(Cp)_2_{[Si(SiMe_3_)_2_SiMe_2_]_2_}]	**30-Tm**	2.9733(21)^a^	53
[K(18-crown-6)(THF)]_2_[{Ce(Cp)_3_}_2_{μ-Si(SiMe_3_)_2_SiMe_2_}]	**31**	3.228(2)	53
[K(2.2.2-crypt)][Y(C_5_H_4_Me)_3_(SiH_2_Ph)]	**32**	2.9531(7)	68
[K(18-crown-6)][Ho(Cp)_3_{Si(SiMe_3_)_3_}]	**33-Ho**	3.022(6)	67
[K(18-crown-6)][Tm(Cp)_3_{Si(SiMe_3_)_3_}]	**33-Tm**	3.018(2)	67
[{K(18-crown-6)}_2_Cp][Ce(Cp)_3_{Si(SiMe_3_)_3_}]	**34-Ce**	3.155(5)	67
[{K(18-crown-6)}_2_Cp][Sm(Cp)_3_{Si(SiMe_3_)_3_}]	**34-Sm**	3.1031(17)	67
[{K(18-crown-6)}_2_Cp][Gd(Cp)_3_{Si(SiMe_3_)_3_}]	**34-Gd**	3.067(3)	67
[{K(18-crown-6)}_2_Cp][Tm(Cp)_3_{Si(SiMe_3_)_3_}]	**34-Tm**	3.014(2)	67
[Y(Cp)_3_{Si[{N(CH_2_^*t*^Bu)}_2_C_6_H_4_-1,2]}]	**35-Y**	3.038(2)	69
[Yb(Cp)_3_{Si[{N(CH_2_^*t*^Bu)}_2_C_6_H_4_-1,2]}]	**35-Yb**	2.984(2)	69
[U(Cp)_3_(SiPh_3_)]	**36**	––b	72
[U(Cp)_3_{Si(SiMe_3_)_3_}]	**37**	––b	74
[Th(Cp*)_2_(Cl){Si(SiMe_3_)_3_}]	**38**	––b	75
[Th(Cp*)_2_(Cl)(Si^*t*^BuPh_2_)]	**39**	––b	75
[U{N(^*t*^Bu)C_6_H_3_-3,5-Me_2_}_3_{Si(SiMe_3_)_3_}]	**40**	3.091(3)	76
[U(Cp′)_3_{Si(NMe_2_)[PhC(N^*t*^Bu)_2_]}]	**41**	3.1637(7)	77
[U(Cp′)_3_{Si[PhC(N^i^Pr)_2_]_2_}]	**42**	3.1750(6)	77
[Th(Cp′)_3_{Si(SiMe_3_)_3_}]	**43-Th**	3.1191(8)	80
[U(Cp′)_3_{Si(SiMe_3_)_3_}]	**43-U**	3.0688(8)	80

**M–Ge complexes**			
[Yb(GePh_3_)_2_(THF)_4_]	**44**	3.156(3)	40
[Yb{(GePh_2_GePh_2_)_2_}(THF)_4_]	**45**	3.104(2)	85
[Eu(GePh_3_)_2_(DME)_3_]	**46**	3.3484(3)	86
[Dy(C_5_H_4_^i^Pr)_2_(GePh_3_)(THF)]	**47**	2.981(1)	87

**M–Sn complexes**			
[U(Cp)_3_(SnPh_3_)]	**48**	3.1661(15)	70
[Yb{Sn(CH_2_^*t*^Bu)_3_}_2_(THF)_2_]	**49**	3.216(1)	88
[Yb(SnPh_3_)_2_(THF)_4_]	**50**	3.305(1)	89
[(Ph_3_Sn)Yb(THF)_2_(μ-η:^1^η^6^-Ph)_3_Yb(THF)_3_]	**51**	3.379(1)	90
[Sm{Sn(SnMe_3_)_3_}_2_(THF)_4_]	**52-Sm**	3.394(6)	91
[Yb{Sn(SnMe_3_)_3_}_2_(THF)_4_]	**52-Yb**	3.294(6)	91
[La(Cp)_3_{Sn(2-py^5Me^)_3_Li(THF)}]	**53-La**	3.3175(4)	92
[Yb(Cp)_3_{Sn(2-py^5Me^)_3_Li(THF)}]	**53-Yb**	3.0740(9)	92
[Yb{Sn(2-py^5Me^)_2_La(Cp)_3_}_2_]	**54**	3.3353(6)	93
[U(TREN^TIPS^)(SnMe_3_)]	**55**	3.3130(3)	94
[Dy(Cp*)_2_(SnPh_3_)(THF)]	**56**	3.239(0)	87

**M–Pb complexes**			
[Sm(Cp)_3_{Pb(2-py^6-O*t*Bu^)_3_Li}]	**57-Sm**	3.2656(3)	95
[Eu(Cp)_3_{Pb(2-py^6-O*t*Bu^)_3_Li}]	**57-Eu**	3.2038(3)	95

a Mean bond distance.

b X-ray structure determination not reported.

c Highly disordered crystallographic characterisation.

## (A) Ln(ii)–Si chemistry

Ln(ii) silicon complexes to date are only known with the classical divalent ions Sm, Eu and Yb, as divalent precursors are most readily available for these elements.^37^ However, since 1999 synthetic routes have been developed for solvated LnI_2_ precursors for Tm, Dy and Nd,^38^ and the 2+ oxidation state is now known for all Ln (with the exception of radioactive Pm),^39^ hence an extension of Ln(ii) silicon chemistry to other elements in this series is feasible. Considering that the 2+ oxidation state is considerably underdeveloped for the Ln series compared to Ln(iii) chemistry, complexes containing Ln(ii)–Si bonds are relatively abundant as softer Ln(ii) centres are more amenable to bonding with silicon.^9^ Ln(ii) silicon complexes are organised into three categories herein: (1) Ln(ii) silanide complexes that have their coordination spheres completed by THF; (2) Ln(ii) silanide complexes that are stabilised by supporting ancillary ligands; (3) Ln(ii) silylene complexes.

### A1. Ln(ii) silanide complexes with their coordination spheres completed by THF

**Fig. 2:**
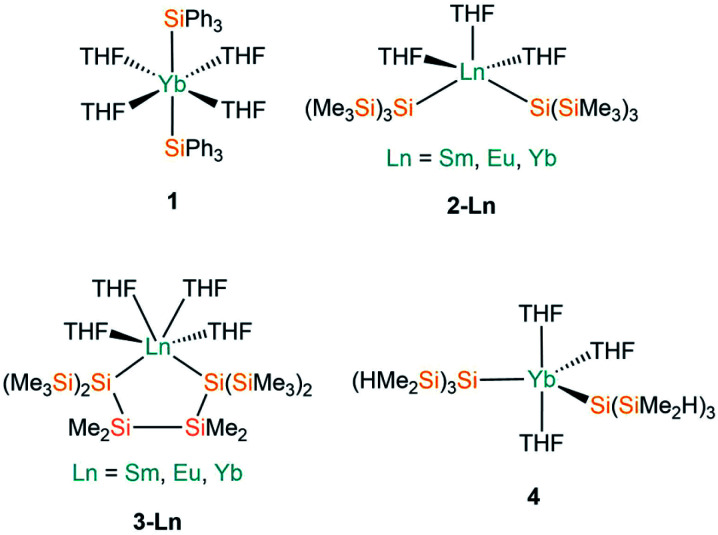
Complexes **1–4**.

THF is the one of the most commonly utilised coordinating solvents in non-aqueous f-element chemistry, particularly as Ln halide precursors are often used as their THF solvates to improve their solubility.^9^ The first example of a divalent lanthanide silanide complex, [Yb(SiPh_3_)_2_(THF)_4_] (**1**, Fig. 2), was reported in 1994 by Bochkarev and co-workers; this is the only example to date of a structurally characterised aryl-substituted silanide Ln complex.^40^ In contrast to the majority of complexes containing Ln–Si bonds prepared to date, **1** was synthesised by the redox reaction of Ph_3_SiCl with an excess of Yb metal at room temperature for one week with concomitant elimination of [YbCl_2_(THF)_2_].

Notable examples derive from the work of Baumgartner, Szilvási and co-workers; in 2015 they synthesised [Ln{Si(SiMe_3_)_3_}_2_(THF)_3_] (**2-Ln**; Ln = Sm, Eu, Yb, Fig. 2)^41^ from the separate salt metathesis reactions of two equivalents of potassium hypersilanide with [LnI_2_(THF)_2_], and they also employed a bidentate derivate of the hypersilanide ligand, {[Si(SiMe_3_)_2_SiMe_2_]_2_}^2−^, to access the chelated *pseudo-cis*-octahedral Ln(ii) cyclopentasilane complexes, [Ln{[Si(SiMe_3_)_2_SiMe_2_]_2_}(THF)_4_] (**3-Ln**; Ln = Sm, Eu, Yb, Fig. 2),^41^ by extending these methodologies. Complexes **3-Sm** and **3-Eu** provided the first structurally characterised examples of Sm–/Eu–silanide complexes that do not feature any supporting ancillary ligands. In addition, this paper also included the first examples of using DFT calculations to probe the nature of the Ln–Si bonds; these computed results supported NMR spectroscopic data in the assignment of a highly shielded anionic silanide fragment.

In 2017 Sadow and co-workers reacted two equivalents of a smaller derivative of potassium hypersilanide, [K{Si(SiMe_2_H)_3_}], with solvated YbI_2_ to afford [Yb{Si(SiMe_2_H)_3_}_2_(THF)_3_] (**4**, Fig. 2).^42^ Complex **4** features β-Si–H groups, which have frequently been employed in silylalkyl and silylamide chemistry, to stabilise the complex with electrostatic interactions between the metal centre and the electron density associated with the β-Si–H bond.

Complexes **2-Ln** and **4** exhibit approximate trigonal bipyramidal geometries, with the two silanide ligands and one THF molecule in the trigonal plane, and the remaining two THF molecules in axial positions. The Si–Ln–Si angles of complexes **2-Ln** do not vary greatly with a change in Ln (mean 123.45(5)°), though the Si–Ln–Si angle for **2-Yb** (124.51°) is significantly smaller than that of **4** (129.69(6)°) due to differences in the size of the silanide ligands. Complexes **3-Ln** exhibit mean Si–Ln–Si angles of 90.727(10)° as a result of the two hypersilanide moieties being tethered together; this results in *pseudo-cis*-octahedral geometries with the remainder of the Ln coordination spheres completed by four THF molecules. Ln–Si bond lengths vary with the decreasing size of Ln radii across the series as expected;^43^ this is particularly apparent for the **2-Ln** and **3-Ln** families, with the Yb–Si bond lengths approximately 0.1 Å shorter than the respective Sm/Eu–Si distances for the same ligand set. The Ln–O distances corresponding to the THF molecules in **1–4** appear to be essentially independent of the identity of the silanide ligand.

**Fig. 3:**
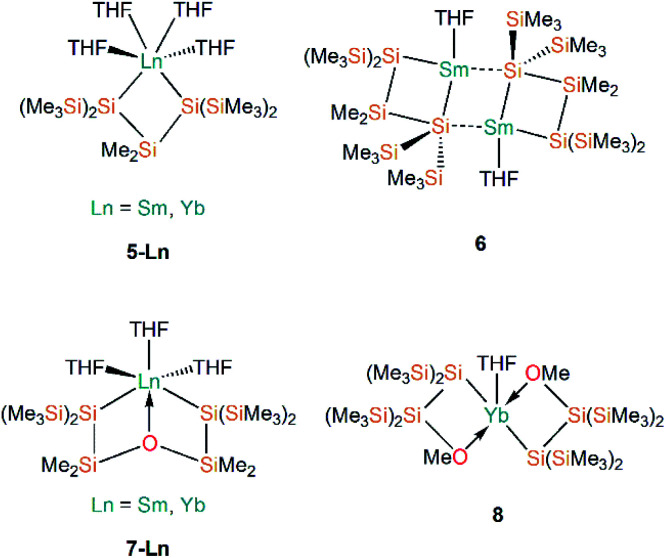
Complexes **5–8**.

Following Baumgartner's initial publication, the same group later targeted solvent-poor silanide Ln(ii) complexes in 2017.^44^ This more recent work utilised a more constrained bidentate ligand to yield the Ln(ii) silanide complexes [Ln{[Si(SiMe_3_)_2_]_2_SiMe_2_}(THF)_4_] (**5-Ln**; Ln = Sm, Yb, Fig. 3); the mean Si–Ln–Si angles in these chelated complexes of 75.48(5)° engender more distorted *pseudo-cis*-octahedral geometries compared to **3-Ln**. Recrystallisation of **5-Sm** in pentane resulted in a dinuclear samarium complex [Sm{Si(SiMe_3_)_2_SiMe_2_}(THF)]_2_ (**6**, Fig. 3); as one of the silanide moieties of each chelating ligand bridge between the Sm(ii) centres of **6** they contain only one bound THF molecule per Sm and these ions exhibit coordination numbers of four.

In the same publication, Baumgartner, Marschner and co-workers also explored functionalised silanide ligands featuring oxygen donor atoms to suppress THF coordination to a greater extent.^44^ Although THF molecules remained coordinated in the products isolated from initial work towards this goal, these complexes exhibited distinctive geometrical features. The bis-silanide ligands in [Ln{[Si(SiMe_3_)_2_SiMe_2_]_2_O}(THF)_3_] (**7-Ln**; Ln = Sm, Yb, Fig. 3) contain Si–O–Si linkages that additionally coordinate the Ln(ii) ions on the same hemisphere as the two silanide groups, providing distorted *fac*-octahedral geometries. The two chelating silyl-ether ligands in [Yb{[Si(SiMe_3_)_2_SiMe_2_]OMe}_2_(THF)] (**8**, Fig. 3) are derived from the addition of a methoxy group at the terminus of the oligosilane. The Yb(ii) centre in **8** exhibits an approximate square-based pyramidal geometry, with a THF molecule occupying the axial position and the Si- and O-donors of the two ligands coordinated in a mutually *trans*-arrangement. The authors concluded in this work that THF could not be readily avoided as a reaction solvent, and that increased steric bulk and coordinating heteroatoms in the ligand scaffold would be required to furnish Ln(ii) silanide complexes that are free of coordinated donor-solvents.^44^

**Fig. 4:**
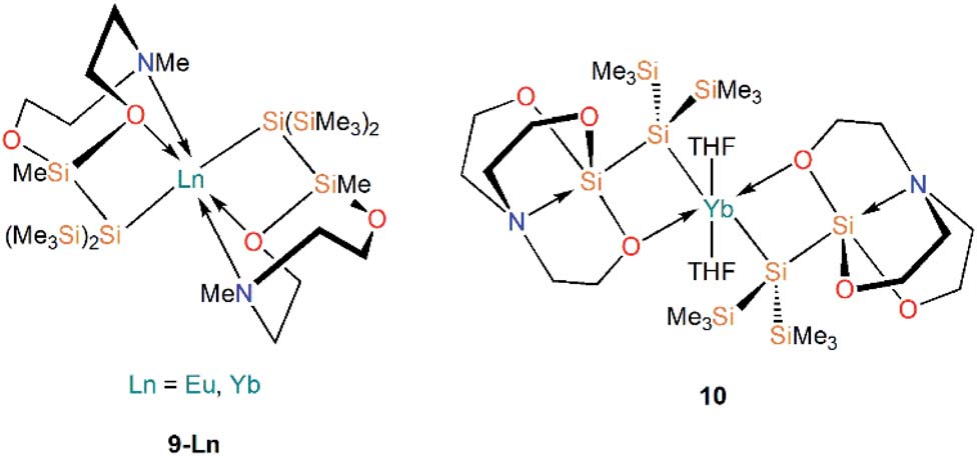
Complexes **9-Ln–10**.

Further work targeting donor solvent-poor or -free complexes in Ln(ii) silanide chemistry saw the development of a multidentate silocanylsilanide ligand, {Si(SiMe_3_)_2_Si(Me)[{OCH_2_CH_2_}_2_NMe]}^−^, which contains four heteroatoms that can potentially donate electron density to metal centres; three of these sites are coordinated to Ln(ii) centres in the first homoleptic donor solvent-free silanide complexes [Ln{Si(SiMe_3_)_2_Si(Me)[{OCH_2_CH_2_}_2_NMe]}_2_] (**9-Ln**; Ln = Eu, Yb, Fig. 4).^44^ Complexes **9-Ln** were prepared by the salt metathesis reactions of two equivalents of the group 1 ligand transfer agent [K{Si(SiMe_3_)_2_Si(Me)[{OCH_2_CH_2_}_2_NMe]}] with the parent [LnI_2_(THF)_2_], where the donor sites in complex **9-Ln** occupy one face of the metal centre. Similarly to their previous work,^41^ Baumgartner, Marschner and co-workers noted that a combination of DFT calculations and ^29^Si NMR spectroscopy support the highly ionic nature of the Ln–Si bond and strong downfield shifted resonances of the metal-bound silicon atoms in **9-Yb** (*δ*_Si_: −182.0 ppm) indicate predominantly silanide character.^44^ Although **9-Ln** were found to be sensitive to visible light, the lack of donor solvent led to a drastic improvement in their stability compared to the solvated complexes reported previously. Around the same time, Baumgartner and co-workers explored differing degrees of electron density on a silatrane-substituted silicon atom and the effect on the Si–N interaction detected by ^29^Si NMR spectroscopy and single crystal XRD analysis.^45^ In this study the authors synthesised a variety of metal silanide complexes, including the Yb(ii) complex [Yb{Si(SiMe_3_)_2_Si([OCH_2_CH_2_]_3_N)}(THF)_2_] (**10**, Fig. 4). One of the silatrane oxygen atoms was found to coordinate to the Yb centre in the solid state structure of **10**, which was in contrast to other complexes presented in this study *i.e.* an analogous reaction of the potassium silyl-silatrane with ZnBr_2_ lead to the formation of a completely linear Si–Zn–Si (180°) arrangement with no Zn–O silatrane interactions and no solvation with THF.^45^

### A2. Ln(ii) silanide complexes stabilised by supporting ancillary ligands

**Fig. 5:**
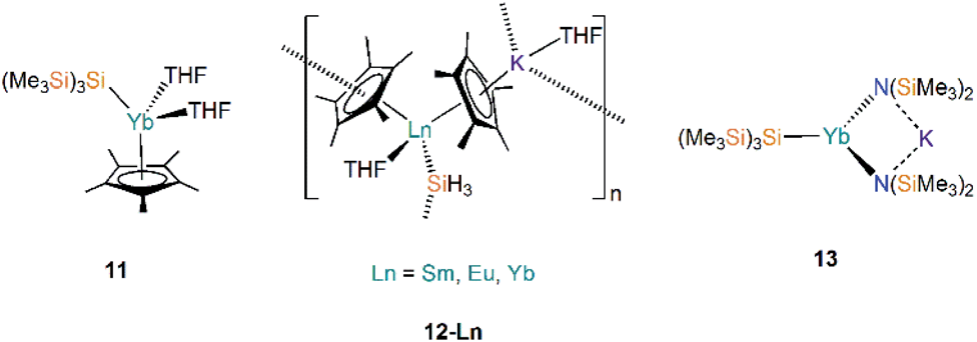
Complexes **11–13**.

There are three examples of Ln(ii) silanide complexes where pre-organised metal coordination spheres were constructed in order to kinetically stabilise the resultant Ln(ii)–Si linkage. In 1996 Corradi *et al.* reacted the sandwich complex [Yb(Cp*)_2_(OEt_2_)] (Cp* = {C_5_Me_5_}^−^) with [Li{Si(SiMe_3_)_3_}(THF)_3_] to yield the mono-cyclopentadienyl complex [Yb(Cp*){Si(SiMe_3_)_3_}(THF)_2_] (**11**, Fig. 5), with elimination of LiCp*.^46^ This study was the first example of Yb–Si coupling observed by both ^29^Si and ^171^Yb NMR spectroscopy, with *J*_Yb–Si_ = 829 Hz. The amount of s-character in the Si sp^3^-hybridised orbital directed at the Yb metal centre primarily determines the strength of coupling; a larger coupling constant is indicative of a stronger, more covalent Yb–Si bond.^46^

In 2003, Hou and co-workers also used Cp* as a supporting ancillary ligand when targeting the synthesis of the phenylsilanide complexes [Ln(Cp*)_2_(SiH_2_Ph){K(THF)_*x*_}] (Ln = Sm, Eu, Yb) through the reaction of parent [Ln(Cp*)_2_(THF)_2_] with *in situ-*generated KSiH_2_Ph. However, as the phenylsilanide reagent was not purified, trace amounts of the Ln(ii)–SiH_3_ complexes, [Ln(Cp*)_2_(SiH_3_){K(THF)}]_*n*_ (**12-Ln**; Ln = Sm, Eu, Yb, Fig. 5), were isolated instead.^47^ Yields of **12-Ln** drastically improved upon the addition of a second equivalent of H_3_SiPh, and independent studies of the synthesis of [KSiH_2_Ph] indicated that [KSiH_3_] is also produced as a major product. Complexes **12-Ln** have shown high activities for the polymerisation of ethylene and styrene, which is postulated to proceed by initial migratory hydrosilylation reactions with these unsaturated hydrocarbons.^47^

In 2006, Niemeyer reported that reaction of [Yb{N(SiMe_3_)_2_}{μ-N(SiMe_3_)_2_}]_2_ with half an equivalent of [K{Si(SiMe_3_)_3_}] yields the solvent-free ‘ate’ complex [Yb{Si(SiMe_3_)_3_}{μ-N(SiMe_3_)_2_}_2_K] (**13**, Fig. 5).^48^ Unlike in the previous example of the synthesis of **11**, there was no elimination of the expected by-product [K{N(SiMe_3_)_2_}] in this reaction. Complex **13** exhibits an approximate trigonal planar geometry about Yb with respect to the silanide and amide donor atoms, and is additionally stabilized by Siβ–Cγ agostic-type interactions from the framework of the silylamide ligand.^48^ The K–N bond distances observed in **13** (2.909(3) Å) are longer than those found in the structurally similar dimer [K{N(SiMe_3_)_2_}]_2_ (2.787(3) Å); from these data Niemeyer suggested the interactions of the silylamide with ytterbium and potassium are competing with a stronger Yb–N interaction observed due to preferential binding to the harder Lewis acid, resulting in longer K–N bonds. The Yb–Si bond length in **13** (3.0387(10) Å) is not significantly different to other reported Yb–Si bond lengths [range: 3.017(4)–3.0644(7) Å],^24^ suggesting the potassium cation plays a spectator role in the formation of **13**.

### A3. Ln(ii) complexes containing silylene ligands

**Fig. 6:**
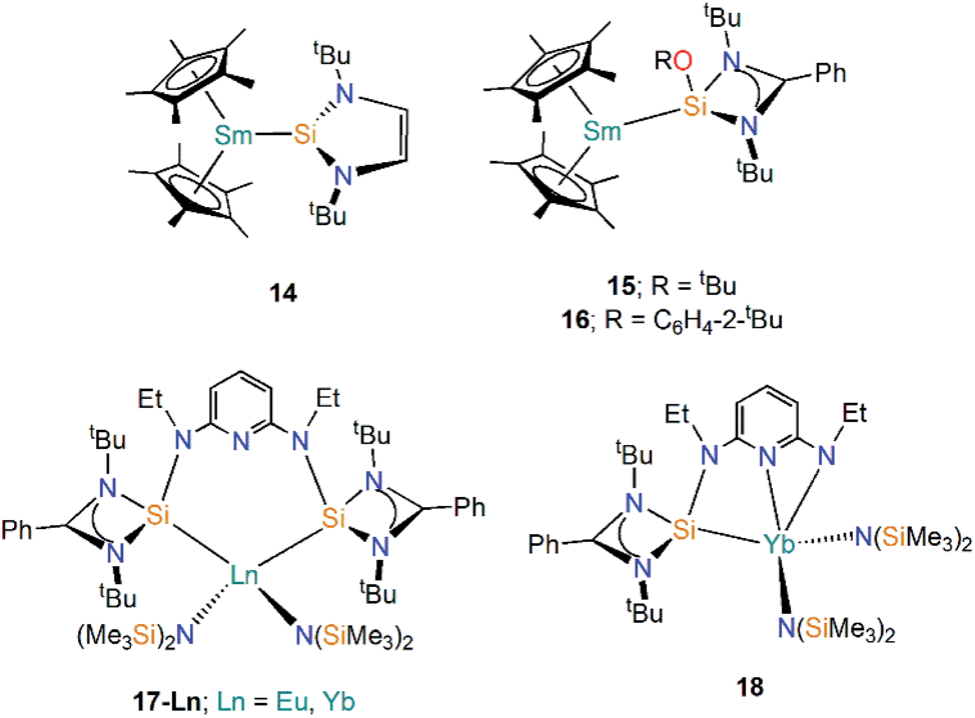
Complexes **14–18-Ln**.

Silylenes (R_2_Si) are silicon analogues of carbenes and are two electron σ-donor ligands that can form adducts with metals through dative bonding.^49^ Analogous to N-heterocyclic carbene (NHC) chemistry, N-heterocyclic silylenes (NHSi) are relatively well-developed due to the push–pull mesomeric-inductive stabilisation mechanism provided by the two N-substituents.^50^ The first example of a divalent lanthanide silylene complex was reported in 2003 by Evans and co-workers, with the reaction of coordinatively unsaturated [Sm(Cp*)_2_] with the NHSi [Si(N^*t*^BuCH)_2_] yielding the Sm(ii) adduct [Sm(Cp*)_2_{Si(N^*t*^BuCH)_2_}] (**14**, Fig. 6).^51^ The dative Si–Sm interaction in **14** is relatively weak and the addition of THF leads to facile displacement of the silylene to give [Sm(Cp*)_2_(THF)_2_] and unbound NHSi. Based on analysis of bond lengths in **14** and the ionic radii of Sm(ii), as well as comparisons with the metrical parameters of the related Sm(ii) NHC complex [Sm(Cp*)_2_{C(NMeCMe)_2_}], the authors suggested that NHSi ligands bind to Sm(ii) centres with a similar bond strength to NHCs,^52^ though there is not always a direct correlation of bond strength and bond length in “long bond” organometallics.^51^

In 2015 Baumgartner investigated Sm(ii) silylene complexes, this time employing an amidinate substituent and a supporting alkoxide or aryloxide substituent to generate the 3-coordinate silylenes [Si(OR){(N^*t*^Bu)_2_CPh}] (R = ^*t*^Bu, C_6_H_4_-2-^*t*^Bu), and adding these reagents to [Sm(Cp*)_2_(OEt_2_)] to give [Sm(Cp*)_2_{Si(OR)[(N^*t*^Bu)_2_CPh]}] (R = ^*t*^Bu, **15**; R = C_6_H_4_-2-^*t*^Bu, **16**, Fig. 6).^53^ These complexes exhibit significantly lower room temperature magnetic moments (**15**: 2.7 *μ*_B_; **16**: 2.6 *μ*_B_) than the starting material [Sm(Cp*)_2_(OEt_2_)] (3.6 *μ*_B_). Although there is a considerable difference in the magnetic moments of **15**, **16**, and the starting material [Sm(Cp*)_2_(OEt_2_)], DFT analyses indicated a predominantly electrostatic donor–acceptor type interaction.^53^

Roesky and co-workers have recently reported the synthesis and reactivity of the bis(silylene)-coordinated Eu(ii) and Yb(ii) complexes [{Ln{SiNSi}{N(SiMe_3_)_2_}_2_] (**17-Ln**; Ln = Eu, Yb, {SiNSi} = {C_5_H_3_N[2,6-{NEt [Si{(N^*t*^Bu)_2_CPh}]}, Fig. 6),^54^ by the addition of one equivalent of {SiNSi} to [Ln{N(SiMe_3_)_2_}_2_(THF)_2_]. The Ln(ii) ions in **17-Ln** adopt distorted tetrahedral arrangements and the long Ln–Si bonds (**17-Eu** = 3.284(2) Å; **17-Yb** = 3.175(2) Å) are indicative of weak metal–silicon interactions, which was verified by the separate addition of d_8_-THF or small nucleophilic carbenes to **17-Yb** resulting in the displacement of the bis(silylene) ligand. Complex **17-Yb** undergoes oxidative thermolysis after heating in toluene for two days to yield the Yb(iii) complex [Yb{C_5_H_3_N-2-NEt,6-{NEt[Si{(N^*t*^Bu)_2_CPh}]{N(SiMe_3_)_2_}_2_] (**18**, Fig. 6); the same process was not observed for **17-Eu** due to the less favourable redox potential of Eu(ii) *vs.* Yb(ii) [Ln^3+^/Ln^2+^; −0.35 V (Eu(ii)), −1.15 V (Yb(ii)) *vs.* NHE].^54^

## (B) Ln(iii)–Si chemistry

Lanthanide chemistry is dominated by the 3+ oxidation state, so it is unsurprising that the genesis of f-element silicon chemistry involved a Ln(iii) complex (see below). However, unlike the complexes covered in Section A1 there are currently no structurally characterised complexes containing Ln(iii)–Si bonds stabilised by external solvent; all examples to date utilise supporting ligands such as halides and, most commonly, cyclopentadienyl ligands. Ln(iii) centres are harder than Ln(ii) centres, so stabilisation with ancillary ligands is of even greater importance here as Ln(iii) centres are less amenable to binding with such soft donor atoms as Si. Complexes in this section are split into three categories; (1) neutral Ln(iii) silanide complexes; (2) charged Ln(iii) silanide complexes; (3) complexes containing silylene ligands.

### B1. Neutral Ln(iii) silanide complexes

**Fig. 7:**
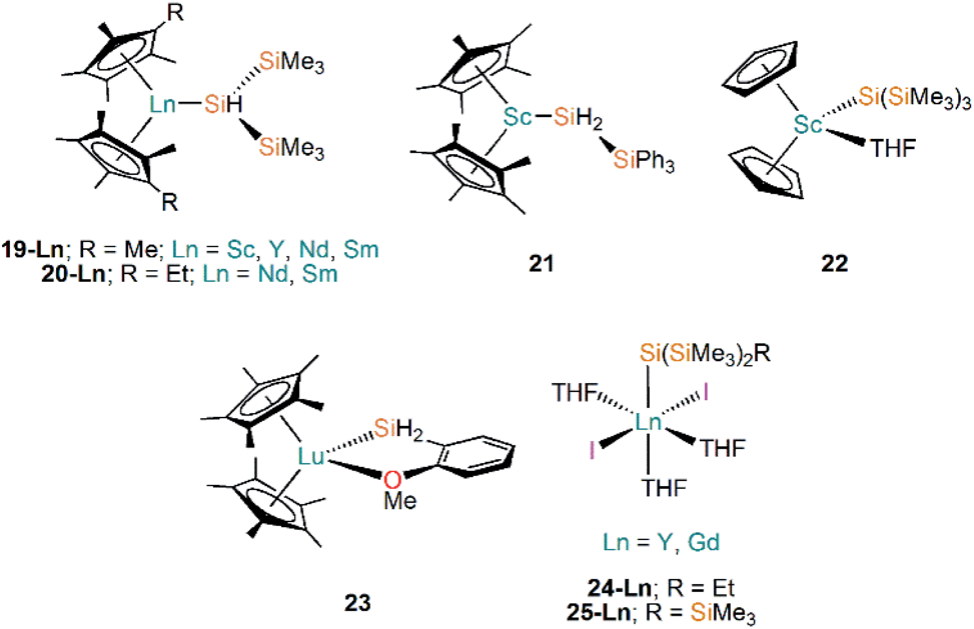
Complexes **19-Ln–25-Ln**.

Following on from the use of cyclopentadienyl ligands to stabilise Ln(ii)–Si bonds (see above), early Ln(iii) silanide work in the 1990s by Tilley and Rheingold exploited alkane elimination strategies to add a secondary silane H_2_Si(SiMe_3_)_2_ at metal centres, yielding [Ln(Cp*)_2_{SiH(SiMe_3_)_2_}] (**19-Ln**; Ln = Y, Nd, Sm, Fig. 7) and [Ln(C_5_Me_4_Et)_2_{SiH(SiMe_3_)_2_}] (**20-Ln**; Ln = Nd, Sm, Fig. 7).^55,56^ A decade later the same authors reported the analogous Sc(iii) complex [Sc(Cp*)_2_{SiH(SiMe_3_)_2_}] (**19-Sc**, Fig. 7), again employing alkane elimination, and extended this chemistry with the use of the primary silanide ligand {SiH_2_(SiPh_3_)} to afford [Sc(Cp*)_2_{SiH_2_(SiPh_3_)}] (**21**, Fig. 7).^57^ In general **19-Ln** and **20-Ln** show high solubilities in non-polar solvents, which made crystallisation challenging; small impurities in the reaction mixture hindered the isolation of a pure material and was shown to result in rapid decomposition of the target compounds. In the case of **19-Nd** a blue-green oil was obtained, but all other complexes in these studies were crystallographically characterised. Reactivity studies of **19-Ln** and **20-Ln** exemplified the reactive nature of the Ln–Si bond, with these complexes undergoing hydrogenation rapidly at 1 atm to produce H_2_Si(SiMe_3_)_2_ and the corresponding lanthanide hydride. In common with the reactivity profile of **12**, **19-Nd** and **19-Sm** were shown to polymerise ethylene with complete consumption of material within 5 minutes. The treatment of **19-Nd** and **19-Sm** with aromatic substituted hydrosilanes, both primary (*e.g.* MesSiH_3_) and secondary (*e.g.* PhMeSiH_2_), lead to the formation of lanthanide hydride complexes [{Ln(Cp*)(μ-H)}_2_].^56^ In later work, Tilley and Sadow investigated why only bulky primary and secondary silanes could be used in alkane elimination reactions to produce Sc–Si bonds, discussed possible mechanisms for the formation of such bonds, and explored the catalytic potential of **19-Sc** and **21** in the alkylation of silanes by σ-bond metathesis.^57^

Subsequently, Tilley and co-workers investigated the reactivity of the first Sc–Si linkage in [Sc(Cp)_2_{Si(SiMe_3_)_3_}(THF)] (**22**, Fig. 7), which was prepared from the reaction of the Sc(iii) dimer [{Sc(Cp)_2_(μ-Cl)}_2_] with two equivalents of [Li{Si(SiMe_3_)_3_}].^58^ Analogous Sc(iii) complexes containing other silanide ligands ({Si(SiMe_3_)_2_Ph}^−^, {Si^*t*^BuPh_2_}^−^ and {SiPh_3_}^−^) were characterised by elemental analysis, and IR and NMR spectroscopy, but no single crystal XRD data was reported for these analogues. Complex **22** was found to polymerise ethylene, but larger alkenes were not oligomerised by this complex; **22** also undergoes carbonylation in a CO atmosphere in 2-methyltetrahydrofuran solution to yield the double insertion product [{Sc(Cp)_2_OC{Si(SiMe_3_)_3_}=C=O}_2_].

Following the same alkane elimination procedures used in the preparation of **19-Ln** and **21-Ln**, in 2001 Castillo and Tilley reported the synthesis of the Lu(iii) complex [Lu(Cp*)_2_{SiH_2_(*o*-MeOC_6_H_4_)}] (**23**, Fig. 7), which contains a rare example of a Lu–Si bond.^59^ Although the authors did not explicitly explore the reactivity of the Lu–Si linkage in **23**, they did investigate the utility of Lu complexes in the hydrogenolysis of organosilanes, including the conversion of phenylsilane into benzene and polysilanes under an atmosphere of dihydrogen; this work concluded that these catalytic processes proceed *via* a Lu⋯Si transition state.^59^ Sgro and Piers reported the synthesis of the Ln(iii) silanide complexes [Ln{Si(SiMe_3_)_2_Et}(I)_2_(THF)_3_] (**24-Ln**, Ln = Y, Gd, Fig. 7) and [Ln{Si(SiMe_3_)_3_}(I)_2_(THF)_3_] (**25-Ln**, Ln = Y, Gd, Fig. 7) in 2014.^60^ Unusually for f-element silicon chemistry, **24-Ln** and **25-Ln** feature halides as ancillary ligands; these distorted octahedral complexes exhibit *mer*-configurations and were found to rapidly decompose when exposed to vacuum, signifying the facile loss of THF and the importance of the saturation of the metal coordination spheres to their stability. Migratory insertion reactions of isocyanide and carbodiimides into the Ln–Si bonds of **24-Ln** and **25-Ln** were performed, exemplifying the reactivity of these linkages and mirroring that which is known for f-element alkyl complexes.^15^ Analysis of the multiplicity of the ^29^Si NMR spectra in diamagnetic **24-Y** and **25-Y** was shown to be a useful tool for monitoring these insertion reactions.

**Fig. 8:**
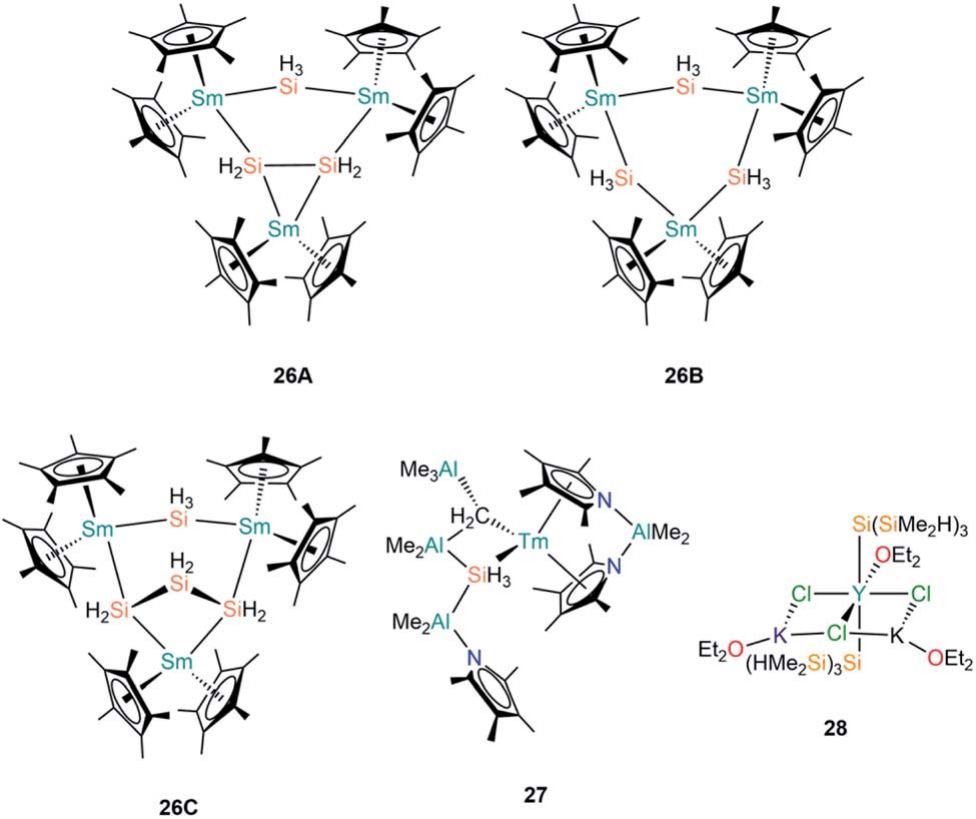
Complexes **26–28**.

In 1996, Tilley and Rheingold reported that reaction of the Sm(iii) alkyl complex [Sm(Cp*)_2_{CH(SiMe_3_)_2_}] with the secondary phenyl silane Ph_2_SiH_2_ produces the trinuclear cluster [Sm_3_Cp*_6_(μ-Si_2_H_4_)(μ-SiH_3_)] (**26A**, Fig. 8) by alkane elimination and silane redistribution, with triphenylsilane (Ph_3_SiH) as a by-product.^61^ On the assumption that all the Sm centres in **26A** remain in the 3+ oxidation state and guided by the Si–Si bond length of 2.458(7) Å, the depiction of **26A** in Fig. 8 is an accurate representation of the product. In contrast, the reaction of [Sm(Cp*)_2_{CH(SiMe_3_)_2_}] with the primary silane PhSiH_3_ furnished a distribution of phenylsilanes as well as three trinuclear clusters: **26A**, [Sm_3_Cp*_6_(μ-SiH_3_)_3_] (**26B**) and [Sm_3_Cp*_6_(μ-Si_3_H_6_)(μ-SiH_3_)] (**26C**); in all of these aggregates the SiH_3_ ligands each bridge two Sm(iii) centres (Fig. 8). Analysis of disorder in the crystal structure determined that the product distribution ratio was 1 : 5 : 4 for **26A** : **26B** : **26C**.^61^ The authors reasoned that the reaction with a less substituted and more hydride-rich silane is the reason for **26B** and **26C** being present in a higher abundance than **26A** in these mixtures. In later work, Tilley and Castillo showed that the addition of hard Lewis bases such as Ph_3_PO and (Me_2_N)_3_PO to reaction mixtures led to the trinuclear aggregates being broken down into mononuclear complexes of the general formula [Sm(Cp*)_2_(SiH_3_)(L)] (L = Lewis base), although no solid state structures were reported; these species presumably contain terminal Sm–SiH_3_ linkages, which were found to be more amenable to reactivity studies, including the 1,2-migratory insertion of benzophenone into the Sm–Si bond.^62^

Gambarotta and Korobkov reported the first structurally characterised example of a Tm(iii)–Si bond in 2009, when [Tm{AlMe_2_(η^5^-NC_4_Me_4_)_2_}(AlMe_3_)(μ-CH_2_)(μ-SiH_3_)(AlMe_2_){AlMe_2_(NC_4_Me_4_)}] (**27**, Fig. 8) was found to form from a Tm–pyrrolide/aluminate complex.^63^ The Tm centre in **27** was assigned a 3+ oxidation state based on the pale colour of the complex. The SiH_3_ unit of the aluminate-based ligand in **27** was disordered over two positions in a ratio of 64 : 36 in the single crystal XRD data, with Tm–Si distances of 3.087(6) Å and 2.573(6) Å in the respective components, indicating that there is some delocalisation of charge in this ligand that engenders a high degree of flexibility in how it binds to the metal.

In the same paper where the synthesis of **4** was disclosed, Sadow and co-workers reported the reaction of YCl_3_ with three equivalents of [K{Si(SiMe_2_H)_3_}] in diethyl ether at −78 °C for 8 hours to afford a polymeric yttrium silanide ‘ate’ complex, [Y{Si(SiMe_2_H)_3_}_2_(OEt_2_)(μ^2^-Cl)_2_(μ^3^-Cl)K_2_(OEt_2_)_2_]_∞_ (**28**, Fig. 8).^42^ The authors found the characterisation of **28** problematic due to its rapid decomposition at room temperature in both solution and the solid state into unidentified silyl-containing species. The solid state structure of **28** features monomeric units with approximately octahedral Y(iii) centres, with *trans*-disposed silanide groups with mean Y–Si bond lengths of 3.035(1) Å, which are significantly longer than the corresponding distances in **24-Y** (2.9613(18) Å) and **25-Y** (2.979(3) Å).^60^ The equatorial plane about the Y(iii) centres in **28** is composed of one molecule of diethyl ether and three chlorides, with the halides bridging to two potassium cations to form a six-membered YCl_3_K_2_ ring; each potassium vertex is capped with a molecule of diethyl ether. The 1D polymeric chain in the solid state structure of **28** is formed by the potassium centres completing their coordination spheres by each bridging to a chloride and one of the H–Si groups of the SiMe_2_H substituents. A low temperature (200 K) ^29^Si NMR spectrum of **28** exhibited a resonance at −141.6 ppm for the Y–Si atoms and a signal at −9.1 ppm for the SiMe_2_H moieties.^42^

### B2. Charged Ln(iii) silanide complexes

**Fig. 9:**
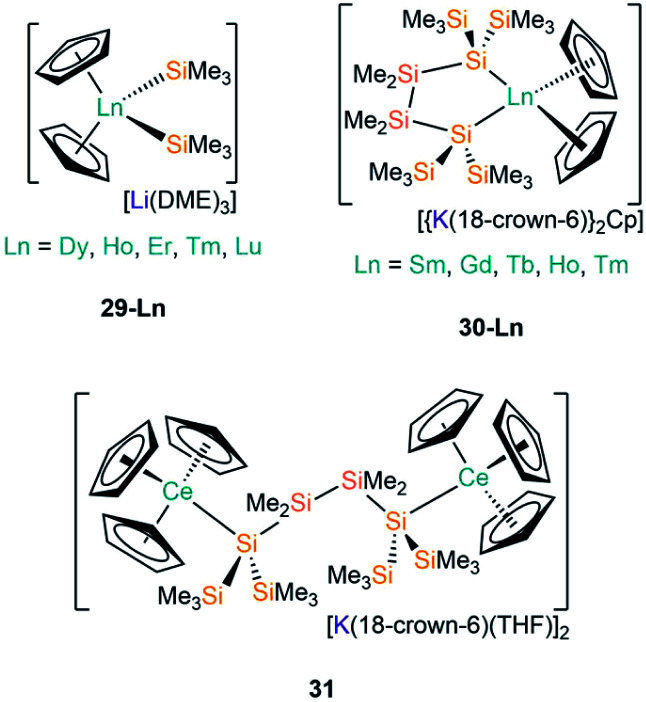
Complexes **29-Ln–31**.

The first report of structurally authenticated Ln–Si bonds was in 1985 by Schumann and co-workers, where they synthesised and characterised [Li(DME)_3_][Lu(Cp)_2_(SiMe_3_)_2_] (**29-Lu**, Fig. 9) from the reaction of [Ln(Cp)_2_Cl(NaCl)(DME)] with two equivalents of [LiSiMe_3_];^64^ this series was later extended to include analogous complexes for Ln = Dy, Ho, Er and Tm (Fig. 9).^65,66^ The solvated ‘ate’ complexes **29-Ln** remain the only examples of Dy–Si and Er–Si bonds reported to date; all are temperature sensitive and slowly decompose at room temperature, even under an argon atmosphere, with rapid decomposition occurring when samples were heated above 75 °C.

The second family of charged Ln(iii) complexes containing Ln–Si bonds was reported 30 years after Schumann's first report; in 2015 Baumgartner disclosed the ligand scrambling and rearrangement of tris-cyclopentadienyl lanthanide complexes with the same bidentate oligosilanylsilanide potassium ligand transfer agent used in the synthesis of **3-Ln** (see above) to yield [{K(18-crown-6)}_2_Cp][Ln(Cp)_2_{[Si(SiMe_3_)_2_SiMe_2_]_2_}] (**30-Ln**; Ln = Gd, Tb, Ho, Tm, Fig. 9).^53^ In the same work the authors carried out an analogous reaction with CeCp_3_, however, unlike for the heavier analogues, the acyclic complex [K(18-crown-6)(THF)]_2_[{Ce(Cp)_3_}_2_{μ-Si(SiMe_3_)_2_SiMe_2_}_2_] (**31**, Fig. 9) formed, where the oligosilanylsilanide ligand bridges between two {CeCp_3_} termini, and the two [K(18-crown-6)(THF)]^+^ cations balance the overall charge.^53^ Complexes **30-Gd**, **30-Tb** and **31** respectively contain the first examples in the literature of Gd–Si, Tb–Si and Ce–Si bonds. There is a strong correlation of the Ln–Si bond length decreasing with Ln ionic radii across the Ln series.^43^ DFT calculations indicated that the amount of covalency in the Ln–Si bond also decreases across the Ln series; although the bonding is predominantly ionic in all cases, less orbital extension of the silicon sp^3^-hybridized lone pair towards the metal was suggested for the smaller Ln.^53^ In a later report, Baumgartner and co-workers reported a samarium analogue **30-Sm** to add to the **30-Ln** series.^67^

**Fig. 10:**
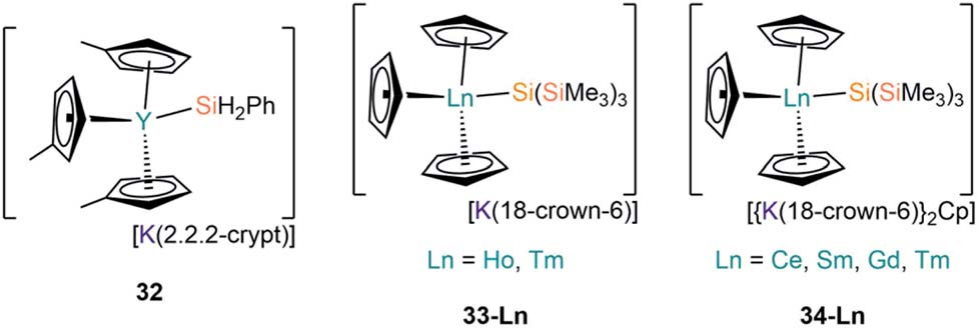
Complexes **32–34-Ln**.

In 2018, Evans and co-workers investigated the reduction of a Y(iii) complex [Y(C_5_H_4_Me)_3_] with K in the presence of 2.2.2-cryptand to generate the Y(ii) anion [Y(C_5_H_4_Me)_3_]^−^, and added phenylsilane to this reaction mixture in efforts to open up new reactivity patterns of non-traditional Ln(ii) species.^68^ Although the authors anticipated the formation of a yttrium hydride complex, they were able to crystallise [K(2.2.2-crypt)][Y(C_5_H_4_Me)_3_(SiH_2_Ph)] (**32**, Fig. 10).^68^ Evans and co-workers attempted to characterise **32** by ^1^H NMR spectroscopy, but they could not identify the –SiH_2_Ph moiety, instead observing resonances associated with {C_5_H_4_Me} as well as a triplet signal with a coupling constant of 34.3 Hz, which is characteristic of a bridging hydride. By repeating the reaction with an excess of phenylsilane colourless crystals of the expected complex [Y(C_5_H_4_Me)_2_(μ-H)]_2_ were isolated. The authors postulated that **32** may decompose in solution at room temperature into the bridging hydride complex.^68^

Most recently, Baumgartner, Marschner and co-workers performed the reactions of LnCp_3_ (Ln = Ce, Sm, Gd, Ho, Tm) with [(18-crown-6)K{Si(SiMe_3_)_3_}].^67^ For Ln = Ho and Tm [K(18-crown-6)][Ln(Cp){Si(SiMe_3_)_3_}] (**33-Ln**; Ln = Ho, Tm, Fig. 10) were isolated, however, in the case of Tm, crystals of [{K(18-crown-6)}_2_Cp][Tm(Cp)_3_{Si(SiMe_3_)_3_}] (**34-Tm**, Fig. 10) were also observed. For Ln = Ce, Sm and Gd **34-Ln** was isolated exclusively. The formation of **34-Ln** for all the Ln investigated except Ho indicates that this is the favoured product of these reactions, where a second equivalent of both starting materials is required to provide the extra {K(18-crown-6)Cp} moiety found in the cation. EPR spectroscopy was attempted on samples of **33-Ln** and **34-Ln** but the large spin–orbit interaction coupled with short relaxation times precluded interpretable spectra in the temperature regimes investigated. NMR spectroscopy of these paramagnetic complexes also proved difficult to interpret due to concentration-dependent chemical shifts.^67^

### B3. Ln(iii) complexes containing silylene ligands

**Fig. 11:**
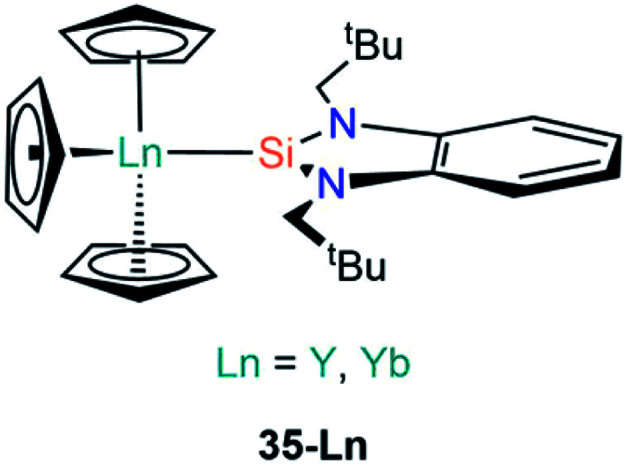
Complexes **35-Ln**.

To date there are only two structurally characterised examples of Ln(iii) silylene complexes; the adducts [Ln(Cp)_3_{Si[{N(CH_2_^*t*^Bu)}_2_C_6_H_4_-1,2]}] (**35-Ln**; Ln = Y, Yb, Fig. 11) were both reported by Lappert and co-workers in 2011.^69^ In common with the complexes discussed in Section A3, the silylene ligands in **35-Ln** dissociate in solution; this was monitored for the Y analogue by variable temperature ^29^Si NMR spectroscopy studies, which showed complete dissociation at 338 K. A *J*_Y–Si_ coupling constant of 59 Hz was found for **35-Y**, which is of a comparable magnitude to *J*_Y–C_ coupling constants for yttrium alkyl complexes, thus the authors suggested that there is some orbital overlap in the Y–Si bond. Lappert and co-workers noted that as [Y(Cp)_3_] and [La(Cp)_3_] are polymeric chains in the solid state, the breaking of these chains is required to form the monomeric complexes **35-Ln**, thus they postulated in the case of La the energy required to rupture the polymeric chain is too high for the silylene to overcome. The authors concluded that Ln–Si bond formation in this case is not dependent on the size of the metal but is instead based upon the ability of the silylene binding to overcome the depolymerisation term associated with the [Ln(Cp)_3_] starting material.

## (C) An–Si chemistry

In comparison to the Ln series, the silicon chemistry of Ans has developed very slowly. Practical issues related to radiological hazards limits the number of researchers working with naturally occurring uranium and thorium, and the hazard and scarcity of synthetic transuranic elements (Np, Pu, Am, *etc*…) requires specialist facilities, hindering the development of their chemistry further.^25^ There are only five crystallographically authenticated examples of complexes exhibiting An–Si bonds to date, however, there have been several other reports of complexes containing An–Si bonds that have been characterised by other techniques. Therefore, this section will be arranged into the following two categories; (1) non-crystallographically authenticated examples of An–Si complexes; (2) crystallographically authenticated An–Si complexes.

### C1. Non-crystallographically authenticated examples of An–Si complexes

**Fig. 12:**
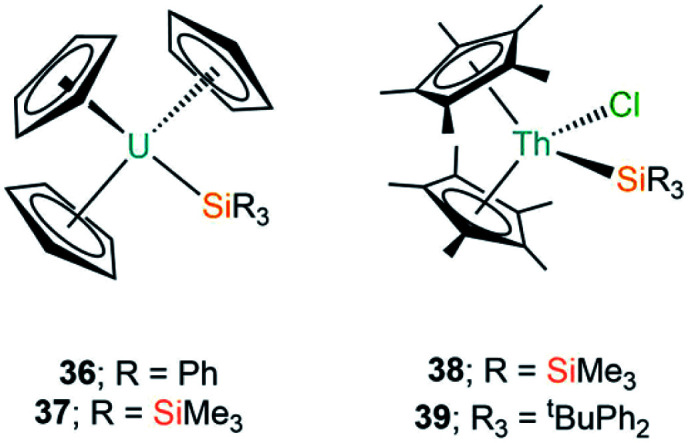
Complexes **36–39**.

The first report of a complex exhibiting an An–Si bond was in 1989 by Porchia and co-workers, which followed previous reports of germanium and tin analogues (see Section D).^70–72^ In the silanide work the authors reacted [U(Cp)_3_Cl] with a freshly prepared sample of [LiSiPh_3_] at low temperature in THF. After 30 minutes the reaction was worked up to yield a brown-green powder, which was identified as the U(iv) complex [U(Cp)_3_(SiPh_3_)] (**36**, Fig. 12) by consideration of elemental analysis and mass spectrometry data.^72^ The authors noted that if the synthesis of **36** was attempted with the potassium salt [KSiPh_3_] then a mixture of products was obtained, and if the reaction mixture was warmed to room temperature in ethereal solvents the main product formed was [U(Cp)_3_(OSiPh_3_)]. The IR spectrum of **36** exhibits vibrational band positions and intensities that are comparable to previously reported germanium and tin homologues,^70,71^ indicating that the uranium centre has a similar coordination environment and further supporting the formulation of **36**. An interesting reactivity profile of **36** is the σ-bond metathesis reaction with HSnPh_3_ to yield [U(Cp)_3_(SnPh_3_)], with the thermodynamic driving force being the greater p*K*_a_ of the Sn–H bond *versus* the Si–H bond formed. In a follow up report Nolan *et al.* investigated the thermochemistry of **36** through ^1^H NMR spectroscopy titrations and iodinolytic calorimetry experiments, through which they determined a bond dissociation energy of 35(4) kcal mol^−1^ for the U–Si bond, indicating that this linkage is relatively unstable and is prone to displacement or insertion of oxygen, as had already been evidenced in the initial work.^73^

In continued efforts to explore the energetics of metal–silicon bonds, in 1995 Marks and King synthesised a variety of group 4 and f-element silicon complexes and collected thermochemical data to provide insights on the bonding and reactivity of these complexes.^74^ Within this report they synthesised [U(Cp)_3_{Si(SiMe_3_)_3_}] (**37**, Fig. 12) from the salt metathesis reaction of [U(Cp)_3_Cl] with [(THF)_3_Li{Si(SiMe_3_)_3_}] in diethyl ether. Elemental analysis and ^1^H NMR spectroscopy both supported the formulation of **37**; the bond dissociation energy calculated for **37** was determined to be 37(3) kcal mol^−1^, indicating that the U–Si linkage in this complex is stronger than the corresponding bond in **36**.

Also in 1995, Tilley and Rheingold reported the double insertion reaction of carbon monoxide into Th–Si bonds.^75^ The authors first reacted [Th(Cp*)_2_(Cl)_2_] with [(THF)_3_Li{Si(SiMe_3_)_3_}] to give [Th(Cp*)_2_(Cl){Si(SiMe_3_)_3_}] (**38**, Fig. 12), however, this yellow complex readily decomposed into [Th(Cp*)_2_(Cl)_2_], HSi(SiMe_3_)_3_ and other products. As a result of this instability **38** was only characterised by ^1^H NMR spectroscopy, but when it was formed in a pressurised CO atmosphere carbonylation occurred to yield the silylated ketene product [Th(Cp*)_2_(Cl)(OC{Si(SiMe_3_)_3_}=C=O)], which could be isolated and identified by single crystal XRD. As carbonylation reactions are well known in d-transition metal chemistry the formation of a thorium ketene in this reaction is additional evidence that a Th–Si bond was present in the proposed intermediate **38**. Analogous results were also observed for the mixed aryl-alkylsilanide ligand {Si^*t*^BuPh_2_}, with [Th(Cp*)_2_(Cl)(Si^*t*^BuPh_2_)] (**39**, Fig. 12) proposed as an intermediate.^75^

### C2. Crystallographically authenticated An–Si complexes

**Fig. 13:**
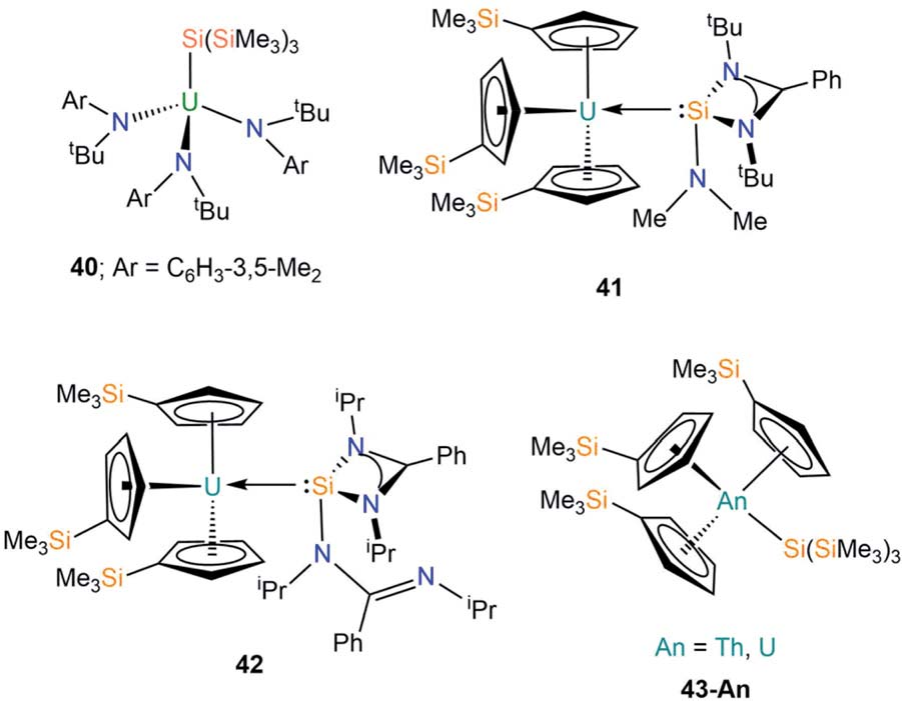
Complexes **40–43-An**.

In 2001 Cummins and co-workers reported the first crystal structure of an actinide silicon complex, with the salt metathesis reaction of [U{N(^*t*^Bu)Ar}_3_(I)] (Ar = C_6_H_3_-3,5-Me_2_) with [(THF)_3_Li{Si(SiMe_3_)_3_}] yielding [U{N(^*t*^Bu)Ar}_3_{Si(SiMe_3_)_3_}] (**40**, Fig. 13).^76^ Complex **40** has a U–Si bond length of 3.091(3) Å, but as the first crystallographically characterised molecular U–Si complex no meaningful comparisons could be made with the metrical parameters of other actinide complexes. As a result, DFT calculations were performed on the model complexes [U(NH_2_)_3_(EH_3_)] (E = C, Si, Ge, Sn), with a calculated U–Si bond length of 2.992 Å in the silicon analogue indicating that steric buttressing of the *tert*-butyl amide and the trimethylsilyl groups in **40** result in a longer U–Si bond than the computational model. The authors reported a U–Si bond energy of 45 kcal mol^−1^, which is larger than previous reports (see Section C1), reinforcing the increased solution stability of **40** at room temperature over previous examples. Complex **40** was found to be surprisingly reticent to participate in insertion chemistry, with no reactions observed with reagents such as CO, isocyanates and isonitriles.

In 2020, Arnold and co-workers reported the synthesis and solid state structures of two U(iii)–silylene complexes.^77^ As [U(Cp′)_3_] (Cp′ = C_5_H_4_SiMe_3_) had previously been used as a starting material to generate dative U(iii)–E(I) bonds (E = Al, Ga),^78^ and [U(Cp′)_3_] was the first molecular uranium complex shown to bond with CO at room temperature,^79^ this starting material was selected to stabilise the first structurally characterised examples of actinide heavy tetrylene complexes. The separate reactions of the amidinate-supported silylenes [Si{PhC(N^*t*^Bu)_2_}(NMe_2_)] and [Si{PhC(N^i^Pr)_2_}_2_] with [U(Cp′)_3_] provided the first examples of U(III)–Si complexes, [U(Cp′)_3_{Si(R)[PhC(NR′)_2_]}] (**41**; R = NMe_2_, R′ = ^*t*^Bu, **42**; R = PhC(N^i^Pr)_2_, R′ = ^i^Pr; Fig. 13).^77^ Complexes **41** and **42** have U–Si bond lengths of 3.1637(7) and 3.1750(6) Å, respectively. As a result of containing a bulkier silylene, one of the amidinates bound to silicon in **42** has switched to a monodentate binding mode upon coordinating to uranium to minimise steric strain (see Fig. 13). No signal was observed in the ^29^Si NMR spectra of **41** and **42** for the silicon atoms bonded to uranium, but these spectra revealed that the Cp′ silicon signal was largely unchanged from the starting material. These data, coupled with relatively strong f–d transitions in the visible region of UV-Vis-NIR spectra indicate a U(iii) oxidation state in **41** and **42**. Moreover, for **42** the UV-Vis-NIR spectrum is largely the summation of the individual starting materials, whereas FTIR spectroscopy indicates that the U–Si bond is intact in the solid state, suggesting that in solution **42** is in a dynamic equilibrium. These data imply that the strength of the U–Si bonding interactions in **41** and **42** is strongly dependent upon the steric effects of the silylene used. DFT calculations support the experimental evidence of weak U–Si interactions in **41** and **42**, with respective bond dissociation energies of 11.9 and 6.9 kcal mol^−1^ determined. Complex **41** was found to have a low-lying bonding molecular orbital for the U–Si bond, whereas this is more accessible for **42** and is therefore more easily perturbed. The U–Si interactions in both complexes are best described as polarised σ-bonding, with the second-order NBO level revealing a significant π-back bonding component in **41**, which is not as prevalent in **42**.

We have recently contributed to the field of actinide silicon chemistry with the synthesis of the structurally analogous Th and U silanide complexes [An(Cp′)_3_{Si(SiMe_3_)_3_}] (**43-An**, An = Th, U, Fig. 13) by the salt metathesis reactions of parent [An(Cp′)_3_Cl] with one equivalent of [K{Si(SiMe_3_)_3_}].^80^ The isolation of **43-An** provided the first structurally authenticated Th–Si bond, allowing a meaningful comparison of the An–Si bonds of Th(iv) and U(iv) ions; the An–Si bond lengths of 3.1191(8) Å (**43-Th**) and 3.0688(8) Å (**43-U**) revealed the shortest U–Si bond reported to date. Additionally, we reported the first ^29^Si NMR spectral chemical shifts of actinide-bonded silicon atoms for **43-An** [**43-Th** = −108.92 ppm; **43-U** = −137.09 ppm]. Quantum chemical calculations revealed strongly polarised single An–Si σ-bonds, with largely similar 7s/6d/5f An contributions to the An–Si bonds for Th and U; these were quantified by the QCT interatomic exchange–correlation energy, *V*_XC_, to provide a covalency metric for the An–Si interaction of −0.092 and −0.096 for **43-Th** and **43-U** respectively, which agree with both NBO-based metrics and delocalisation indices. The An–Si interactions in **43-An** were found to be kinetically stable in the solid state and in solution for a range of non-aqueous solvent systems, which was attributed to the strong polarised covalent An–Si bonds between the actinide ion and the hypersilanide ligand.^80^

## (D) Germanium, tin & lead

There have only been a handful of reports of f-element complexes containing bonds with heavier tetrels, thus their chemistry is far less developed than silicon. To date no structurally authenticated examples of actinide germanium complexes have been reported, though [U(Cp)_3_(GePh_3_)] has been synthesised and characterised by elemental analysis and mass spectrometry.^71^ All four examples of lanthanide germanium complexes to date are exclusively limited to phenyl substituted ligands; additionally, only three different lanthanides been employed in these studies. For tin there are multiple examples which span a number of ligand types including stannanide and stannylene across both lanthanides and actinides, including a number of Zintl clusters reported in recent years.^81,82^ Finally, there are only two reports of molecular f-block plumbylene complexes, however again there are a number of reports of lead Zintl clusters.^83,84^ This section will summarise molecular examples of f-element complexes of the heavier tetrels in separate sections.

### D1. Germanium

**Fig. 14:**
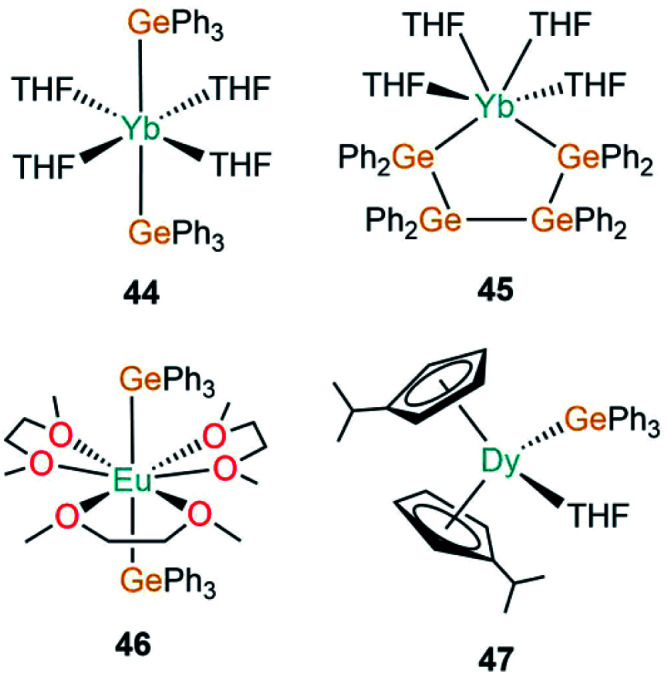
Complexes **44–47**.

The first structurally authenticated example of an f-block germanium complex was disclosed in 1994 by Bochkarev and co-workers in the same report as the first lanthanide(ii)–silanide complex (**1**).^40^ The authors reported the synthesis of [Yb(GePh_3_)_2_(THF)_4_] (**44**, Fig. 14), in the same manner as **1**, complex **44** was synthesised by the redox reaction of Ph_3_GeCl with an excess of Yb metal at room temperature for 11 days in THF with concomitant elimination of [YbCl_2_(THF)_2_].^40^ The mean Yb–Ge bond length of **44** (3.156(3) Å) is statistically identical to the analogous Yb–Si bond length in **1** (3.158(2) Å). Finally, Bochkarev and co-workers disclosed an example of the first and only organogermanium metallacycle complex of the f-block, [Yb{(GePh_2_GePh_2_)_2_}(THF)_4_] (**45**, Fig. 14), from a similar synthesis as reported for **44**; the redox reaction of Ph_2_GeCl_2_ with an excess of ytterbium metal in THF for one week yielded **45** with concomitant elimination of [YbCl_2_(THF)_2_].^85^ The *cis*-arrangement of the ytterbium-bound germanium atoms in **45** rival the *trans*-arrangement found in **44**, with a shorter Yb–Ge bond length seen in the metallacycle (3.104(2) Å).^85^

In 1999, Schumann and co-workers reported the synthesis and structure of [Eu(GePh_3_)_2_(DME)_3_] (**46**, Fig. 14) from the reaction of [Eu(C_10_H_8_)(THF)_2_] with two equivalents of Ph_3_GeH liberating dihydrogen as a by-product to yield **46**.^86^ Additionally, the synthesis of the mono-germanide complex [Eu(GePh_3_)(i)(DME)_2_] was reported from the reaction of C_10_H_8_[EuI(DME)_2_]_2_ with two equivalents of Ph_3_GeH, although the product was found to be unstable in solution and rapidly disproportionated to **46** and [EuI_2_(DME)_2_], precluding structural elucidation.^86^ In 2019, Gao and co-workers reported the synthesis of [Dy(C_5_H_4_^i^Pr)_2_(GePh_3_)(THF)] (**47**, Fig. 14) from the salt metathesis reaction of the separated ion pair [Dy(C_5_H_4_^i^Pr)_2_(THF)_2_][BPh_4_] with KGePh_3_.^87^ Complex **47** showed slow magnetic relaxation at zero field with a barrier to magnetisation reversal of 485 K and a hysteresis temperature of 6 K. DFT calculations performed on a model of **47** suggest a significant amount of covalency in the Dy–Ge bond in comparison to lanthanide alkyl complexes, which are predominantly ionic in nature.^87^

### D2. Tin

**Fig. 15:**
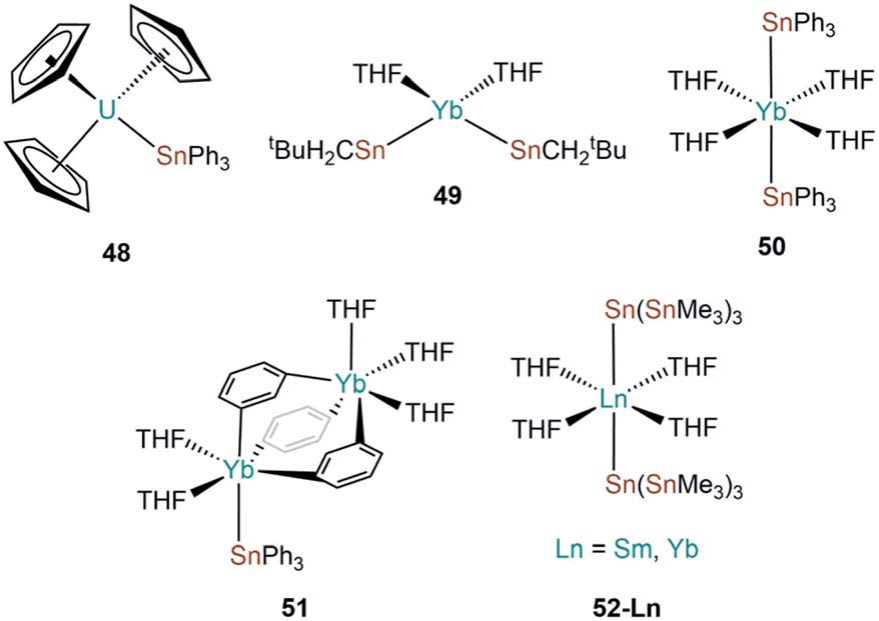
Complexes **48–52-Ln**.

The genesis of f-block tin chemistry was in 1986, when Porchia and co-workers reported the synthesis of [U(Cp)_3_(SnPh_3_)] (**48**, Fig. 15) from the protonolysis reaction of [U(Cp)_3_(NEt_2_)] with HSnPh_3_.^70^ Interestingly, **48** was the only uranium tetrel complex that the authors could crystallise, and analogous triphenylsilanide (**36**)^72^ and triphenylgermanide ([U(Cp)_3_(GePh_3_)])^71^ complexes were solely characterised by elemental analysis and mass spectrometry. The first report of a lanthanide stannanide complex was reported in 1991 by Cloke, Lawless and co-workers, where the tetrahedral complex [Yb{Sn(CH_2_^*t*^Bu)_3_}_2_(THF)_2_] (**49**, Fig. 15) was prepared by the salt metathesis reaction of *in situ*-generated [K{Sn(CH_2_^*t*^Bu)_3_}] and [YbI_2_].^88^ The authors reported the ^171^Yb NMR spectrum of **49**, with *δ*_Yb_ = 725 ppm and both ^119^Sn and ^117^Sn satellites with coupling constants of 8627 and 8254 Hz, respectively; the larger *J*_YbSn_ coupling constant was also observed by ^119^Sn NMR spectroscopy (*δ*_Sn_ = −95 ppm).^88^ Also in 1991, Bochkarev and co-workers reported the synthesis of [Yb(SnPh_3_)_2_(THF)_4_] (**50**, Fig. 15) synthesised from the reaction of [Yb(C_10_H_8_)(THF)_2_] with one equivalent of Ph_4_Sn for two days.^89^ Complex **50** is isostructural to the lighter group 14 congeners **1** and **44**, with a Yb–Sn bond length of 3.305(1) Å consistent with the increase in the covalent radii upon descent of group 14.^89^ Later that same year, Bochkarev reported a second, more unusual product, from the reaction of [Yb(C_10_H_8_)(THF)_2_] and Ph_4_Sn: a bimetallic ytterbium complex with a triphenyltin cap, [(Ph_3_Sn)Yb(THF)_2_(μ-η^1^:η^6^-Ph)_3_Yb(THF)_3_] (**51**, Fig. 15).^90^ The authors postulated that **51** formed *via* the association of *in situ*-generated “[Yb(SnPh_3_)(Ph)(THF)_*n*_]” with “[Yb(Ph)_2_(THF)_3_]” to alleviate steric unsaturation. The Yb–Sn length in **51** is 3.379(1) Å, which is longer than the mean Yb–Sn distance previously reported for **50** (3.305(1) Å).^89^ The mechanism of formation of **51** was proposed by the authors to proceed by a 2 electron transfer from [Yb(C_10_H_8_)(THF)_2_], generating neutral C_10_H_8_ and reductively cleaving one C_Ph_–Sn bond, formally resulting in {Ph_3_Sn} and {Ph} anions and a solvated Yb(ii) cation. Various ligand scrambling processes can occur by Schlenk-type equilibria, with one eventuality leading to complex **50** and another outcome yielding **51**.^90^ In 1993 Bochkarev and co-workers reported the reaction of Me_3_SnCl with either samarium or ytterbium metal in THF for three days to yield [Ln{Sn(SnMe_3_)_3_}_2_(THF)_4_] (**52-Ln**; Ln = Sm, Yb, Fig. 15).^91^ Although a mechanism for this reaction was not provided it should be noted that **52-Yb** can also be produced from the reaction of Me_2_SnCl_2_ and ytterbium metal, suggesting fragmentation of the alkyl-tin bonds and rearrangement occurs to presumably form [ClSn(SnMe_3_)_3_], which can oxidise the lanthanide metal to furnish **52-Ln** and [LnCl_2_(THF)_2_] following ligand scrambling, similar to the formation of **1**.^40,91^

**Fig. 16:**
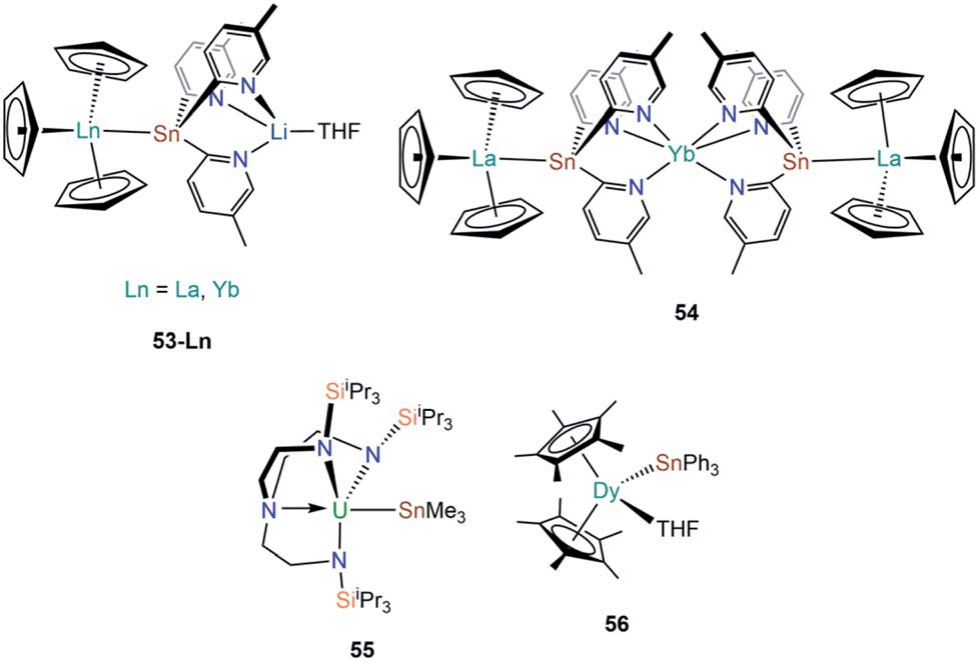
Complexes **53-Ln–56**.

In 2010, Zeckert and co-workers utilised a lithium-stabilised stannylene to form donor–acceptor Ln–Sn bonds with lanthanum and ytterbium tris-cyclopentadienyl complexes to provide [Ln(Cp)_3_{Sn(2-py^5Me^)_3_Li(THF)}] (2-py^5Me^ = 2-C_5_H_3_N-5-Me; **53-Ln**; Ln = La, Yb, Fig. 16).^92^ The authors performed DFT calculations on models of **53-Ln** and proposed that the dative bonds have an appreciable covalent contribution, though should still be considered as predominantly electrostatic. Gratifyingly, the authors were able to record both ^119^Sn and ^139^La NMR spectroscopy of the diamagnetic complex **53-La**, indicating a relatively strong La–Sn interaction that persists in solution, unlike other Ln(iii)–tetrylene complexes (notably **35-Ln**).^69,92^ In subsequent work Zeckert *et al.* showed that the reaction of two equivalents of **53-La** with [Yb(Cp*)_2_(OEt_2_)] yielded [Yb{Sn(2-py^5Me^)_2_La(Cp)_3_}_2_] (**54**, Fig. 16) by a metal displacement reaction, with the concomitant elimination of two equivalents of LiCp*.^93^ The mean La–Sn bond length of **55** (3.3353(6) Å) is longer than found in **53-La** (3.3175(4) Å).^92^ Boncella and co-workers have recently reported a second structurally authenticated uranium–stannanide bond utilising a *N*-silyl-Tren manifold to stabilise the polarising U–Sn linkage (Tren = tris(2-amidoethyl)amine).^94^ Treatment of [U(Tren^TIPS^)Cl] (Tren^TIPS^ = {N(CH_2_CH_2_NSi^i^Pr_3_)_3_}) with [Li{SnMe_3_}] yielded [U(Tren^TIPS^)(SnMe_3_)] (**55**, Fig. 16) by a salt metathesis reaction. Complex **55** was revealed to have a U–Sn bond length of 3.3130(3) Å, which is in stark contrast to the shorter U–Sn distance in **48** of 3.1661(15) Å reported by Porchia,^70^ indicating the steric encumbrance imposed in **55** by the ancillary Tren^TIPS^ ligand. It was noted that **55** decomposed over an extended period of time to eliminate Me_3_SnH and form a U(iv)–cyclometallate, which is a known alternative reaction pathway for many {U(iv)Tren} systems.^12^ DFT calculations of **55** revealed a strongly polarising single U–Sn bond with significant, and directional, contributions from uranium. The paramagnetism of **55** precluded the observation of resonances by ^119^Sn NMR spectroscopy.^94^ In 2019, Gao and co-workers reported the synthesis of [Dy(Cp*)_2_(SnPh_3_)(THF)] (**56**, Fig. 16) alongside the disclosure of **47**; in contrast to the salt metathesis reaction used to synthesise **47**, complex **56** was prepared by the acid–base reaction of [Dy(Cp*)_2_(CH_2_Ph)(THF)] with HSnPh_3_, eliminating toluene as a by-product.^87^ Similar to the analysis of **47**, DFT calculations on a model of **56** were interpreted by the authors to propose a polarised covalent Dy–Sn single bond, and **56** was also found to show single-molecule magnet behaviour with an effective barrier to magnetic reversal of 620 K and a hysteresis temperature of 6 K.^87^

### D3. Lead

**Fig. 17:**
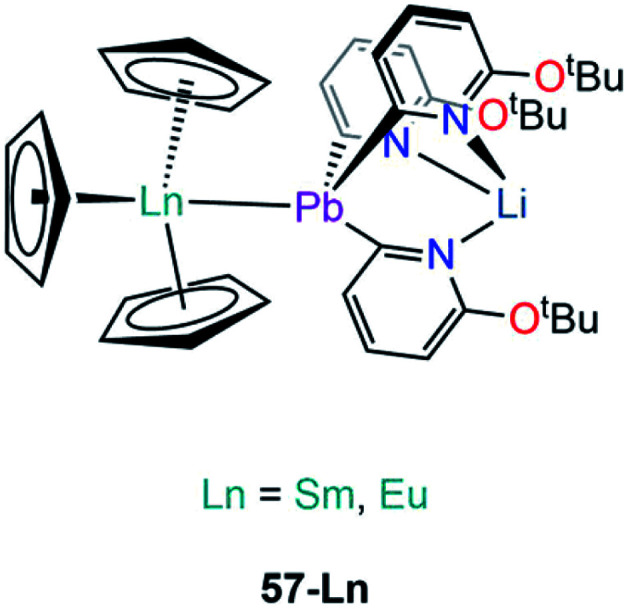
Complexes **57-Ln**.

Besides the two reports of f-block lead Zintl clusters,^83,84^ there is only one report on molecular lead complexes in the f-block by Zeckert and co-workers in 2013, specifically the reactions of a lithium tris(organo)plumbylene [LiPb(2-py^6-O*t*Bu^)_3_(THF)] (2-py^6-O*t*Bu^ = 2-C_5_H_3_N-6-O^*t*^Bu) with [Ln(Cp)_3_] (Ln = Sm, Eu) to form the adducts [Ln(Cp)_3_{Pb(2-py^6-OtBu^)_3_Li}] (**57-Ln**; Ln = Sm, Eu, Fig. 17).^95^ Complexes **57-Ln** exhibit similar motifs to **53-Ln**; Ln–Pb bond lengths of 3.2656(3) Å (**57-Sm**) and 3.2038(3) Å (**57-Eu**) were determined and found to be well within the sum of covalent radii (Alvarez: 3.44 Å).^96^^207^Pb NMR spectroscopy was attempted on **57-Ln** but no signals were observed, and this was attributed to the paramagnetism of Sm(iii) and Eu(iii) ions. Complex **57-Eu** was found to be unstable in solution, undergoing an intramolecular redox reaction resulting in the elimination of a Pb(iii) diplumbane [Pb(2-py^6-O*t*Bu^)_3_]_2_, a Eu(ii) metallocene complex [Eu(Cp)_2_(THF)]_*n*_, and concomitant formation of LiCp.^95^

## Outlook & conclusions

Although the field of f-element silicon chemistry has developed at a relatively steady rate over the last 35 years, reports have started to increase in frequency over the last decade. This can be attributed to the recent renaissance in f-element solution chemistry,^97–99^ with new oxidation states and starting materials becoming more readily available, and the emergence of new motivations for developing the fundamental chemistry of these elements.^9,25^ Throughout the history of f-element silicon chemistry researchers have supported continued progress towards realising the potential of Ln/An–Si bonds through synthetic, spectroscopic and theoretical studies to probe the nature of this linkage; comparative reactivity studies performed with carbon-based analogues has allowed differences and similarities in these bonding regimes to be probed.^51,56,57,59,60,75^ It is clear from this perspective that the tris-(trimethylsilyl)silyl ligand {Si(SiMe_3_)_3_}^−^ and its derivatives constitute the dominant examples of complexes containing Ln/An–Si bonds reported thus far, so an obvious direction for researchers to explore in future is to expand the scope of Si-containing ligands that are utilised in order to provide data on a larger variety of complexes and to allow further benchmarking calculations by computational chemists to be performed.^41,53^ Following the same trend of progress in carbon-based organometallic chemistry, the synthesis and study of Ln/An = Si double bonds is a major target in this area to open up new vistas for future exploration, and to deepen our understanding of the degree of covalency in the predominantly ionic bonding regimes of the f-elements. The f-element chemistry of the heavier tetrels germanium, tin and lead is even less developed and more poorly understood than that of silicon.^26,98^ Although silicon chemistry presents its own unique set of challenges the heavier tetrels will undoubtedly provide new synthetic problems to overcome. We envisage that the parallel development of f-element silicon chemistry with that of the heavier tetrels will provide new and transferable insights to allow more rapid developments in future.

## Conflicts of interest

There are no conflicts to declare.
